# 15-Lipoxygenase and its metabolites in the pathogenesis of breast cancer: A double-edged sword

**DOI:** 10.1186/s12944-021-01599-2

**Published:** 2021-11-27

**Authors:** Mohammad Amin Vaezi, Banafsheh Safizadeh, Amir Reza Eghtedari, Seyedeh Sara Ghorbanhosseini, Mostafa Rastegar, Vahid Salimi, Masoumeh Tavakoli-Yaraki

**Affiliations:** 1 Department of Biochemistry, School of Medicine, Iran University of Medical Sciences, P.O. Box: 1449614535, Tehran, Iran; 2 Department of Biochemistry, Faculty of Pharmacy, Isfahan University of Medical Sciences, Isfahan, Iran; 3 Department of Microbiology, School of Medicine, Golestan University of Medical Sciences, Gorgan, Iran; 4 Department of Virology, School of Public Health, Tehran University of Medical Sciences, Tehran, Iran

**Keywords:** 15-lipoxygenase-1, 15-lipoxygenase-2, Breast cancer, Cell growth, Apoptosis, Metastasis

## Abstract

15-lipoxygenase is one of the key enzymes for the metabolism of unsaturated fatty acids that its manipulation has been proposed recently as a new molecular target for regulating cancer cell growth. Aberrant expression of 15-lipoxygenase enzyme seems to play an indicative role in the pathology of different cancer types, tumor progression, metastasis, or apoptosis. Based on the fact that breast cancer is one of the most common cancers that imposes a burden of mortality in women also, on the other hand, evidence in experimental models and human studies indicate the emerging role of the 15-lipoxygenase pathway in breast cancer pathogenesis, we present a review of recent findings related to the role of 15- lipoxygenase enzyme and metabolites in breast cancer growth, apoptosis, metastasis, and invasion as well as their local and circulating expression pattern in patients with breast cancer. Our review supports the emerging role of 15- lipoxygenase in molecular and cellular processes regulating breast tumor cell fate with both positive and negative effects.

## Metabolism of arachidonic acid, as a long-chain polyunsaturated fatty acid

Omega-3 and omega-6 fatty acids are a family of polyunsaturated fatty acids (PUFA) that play a vital role in the body’s physiological activities due to their presence in cell membranes [1]. Dietary PUFAs include alpha-linolenic acid (ALA), as a short-chain omega-3 fatty acid, and linoleic acid (LA) as a short-chain omega-6 fatty acid, also, eicosapentaenoic acid (EPA), docosahexaenoic acid (DHA), and arachidonic acid (AA) as longer chain omega-3 PUFAs [2]. AA can be produced from the essential fatty acids LA or ALA, direct intake from diet, or release from phospholipid pool by the action of phospholipase A2. The biosynthesis pathway of eicosanoids (bioactive 20-carbon lipid mediators) starts with the conversion of LA to ALA due to the catalytic activity of desaturase. Then, under the influence of the enzyme elongase, this compound is converted to AA, which is a fatty acid with 20 carbon atoms and 4 double bonds [3]. AA can produce eicosanoids through three enzymatic pathways including several oxygenases that generate a spectrum of oxygenated products. One metabolizing way is the cyclooxygenase (COX) pathway, which produces different prostaglandin (PG), such as prostaglandin E (PGE) and PGD, and thromboxane (TX), such as thromboxane A2 (TXA2) and TXA3, and prostacyclins such as prostacyclin I2 and I3 [4]. Two distinct isoforms of the COX enzyme exist, namely COX-1 and COX-2 that are constitutively expressed in most types of cells, although COX-2 is the most regulated type of the COX enzyme that its expression is induced in inflammation and proliferative diseases. Therefore, COX inhibitors appeared to be effective in the treatment of pain, inflammation, stroke, and cancer [2]. The cytochrome P450 (CYP) is the second metabolizing pathway of AA that mainly produces hydroxyeicosatetraenoic acids (HETEs) due to its ω-hydroxylase activity. However, CYP epoxygenase activity resulted in producing epoxyeicosatrienoic acids (EETs) that can further have metabolized by epoxide hydrolase and generate the corresponding diols [5]. Pieces of evidence revealed the vasodilatation effect of EETs as well as their regulatory role on the tumorigenesis process [6]. The last metabolizing way is the lipoxygenase (LOX) pathway that mainly generates leukotrienes (LT), such as LTA4, LTB4, LTC4, LTD4, and LTE4, as well as lipoxins A4, B4, C4, D4, and E4 [7]. LOX pathway is becoming dominant recently as a therapeutic target in many diseases due to its pivotal role in cellular signaling and novel mediators related to this pathway that has been discovered, therefore, the metabolic pathway of LOX is examined in more detail in the next section.

## The role of the 15-LOX metabolizing pathway in the metabolism of polyunsaturated fatty acids

LOX enzymes are a family of iron (non-heme) dioxygenases that can transport oxygen molecules into the free or esterified unsaturated fatty acids. These enzymes are divided into four categories based on the position that the oxygen group enters: lipoxygenase types 5, 8, 12, and 15 [8]. The LOX enzyme uses two major substrates in cells, including AA, a polyunsaturated omega-6 fatty acid (20:4(5,8,11,14)), and LA, a polyunsaturated omega-6 fatty acid (8,2 cis-9,12). The primary product of the enzymatic function of lipoxygenase is hydro peroxy n-6 fatty acids, which are rapidly converted to hydroxy derivatives. Membrane phospholipids are converted to arachidonic acid by phospholipase A4 and phospholipase C, and then AA can be metabolized by lipoxygenase enzymes. The lipoxygenase metabolizes AA and converts it to cyclic hydroperoxides such as hydroperoxy eicosatetraenoicacid (HpETE), Hydroxyeicosatetraenoic acid (HETE), and LTA4. The active metabolites of HpETE are then reduced and converted to HETE. The next substrate for LOX is LA, which is converted to 13-Hydroxyoctadecadienoic acid (13-HODE) [9, 10]. Among all types of lipoxygenases, 15-lipoxygenase (15-LOX), is the target of this study due to its extensive and regulatory roles in cancer pathogenesis. 15-LOX, formerly known as arachidonate lipoxygenase, is made up of two types of isoenzymes: 15-lipoxygenase type 1 (15-LOX-1), and 15- lipoxygenase type 2 (15-LOX-2, 8]. 15-LOX is mainly expressed in reticulocytes, eosinophils and epithelial cells, including respiratory epithelium and macrophages [10]. Intracellular 15-LOX activity is highly regulated and the regulation of this enzyme occurs at the levels of transcription, translation, and post-translation in different cellular systems. The results of previous studies showed that 15-LOX at the transcriptional level can be induced by interleukins [11]. In human monocytes, adenocarcinoma human alveolar basal epithelial cells (A549 cell line), and human colorectal adenocarcinoma cells (Caco2 cell line), 15-LOX expression level was induced following treatment with interleukin 4 (IL4). It was shown that activation of IL4 cell surface receptor, resulted in the activation of signal transducer and activator of transcription 6 (STAT6), and transport of its phosphorylated homodimer to the nucleus to bind to STAT6-responsible agents including IL4-related genes such as 15-LOX [12]. Notably, 15-LOX-1 transcription can be activated both in a STAT6- dependent and independent manner via histone modification of the 15-LOX promotor [13]. Also, it was shown that Interleukin-13 (IL-13) induces the 15-LOX-1 expression and activity that resulted in the elevation of 15-LOX-1 metabolites, activation of peroxisome proliferator-activated receptor-gamma (PPAR-γ), initiation of apoptosis in glioblastoma cells [14]. On the other hand, the transcriptional activity of PPAR*γ* can be facilitated by STAT6 indicating the crucial role of the IL13–15-LOX-PPAR*γ* axis in regulating cell growth and invasion [15]. Besides, it was postulated that 15-LOX expression can be regulated by acetylation of histones in the nucleus [16]. Moreover, Nitric oxide (NO) is assumed to be another regulatory factor for 15-LOX activity, as it was revealed that incubation of 15-LOX for a short time with NO increases the length of the catalytic period of the enzyme, which can cause reversible inhibition of the enzyme [17]. Another important regulatory factor at the post-translational level is the membrane binding ability of 15-LOX in a calcium-dependent manner. When the 15-LOX enzyme was incubated with biological membranes in the presence of calcium ions, the enzyme’s ability to bind to the membrane enhanced that was associated with a 10-fold increase in the enzyme’s catalytic ability to oxidize fatty acids [18]. The 15-LOX the enzyme is involved in various cellular processes, including cell differentiation, development, mitochondrial degradation, and reticulocyte maturation [19–21]. Levels of 15-LOX products (15(*S*)- hydroperoxy eicosatetraenoic acid (15-S-HETE), 3(*S*)-hydroxy-9*Z*,11*E*-octadecadienoic acid (13(*S*)-HODE)) in tissues usually increase during inflammation [22]. However, the role of lipoxygenase enzymes in the development and spread of cancer is very complex and controversial. Evaluating the expression and activity level of lipoxygenases in epithelial cancerous and normal tissues in humans and mice shows that 15-LOX- type 1 and 2 are preferably expressed more in normal /benign tissues than cancerous tissues of the bladder [23], breast [24], colon [25], lung [26] and prostate [19]. However, conflicting evidence exists regarding the 15-LOX expression pattern in the aforementioned tissues. Based on shreds of evidence, LOX isoforms revealed both anti-tumorigenic and pro-tumorigenic effects in different tumor tissues [16, 27]. In breast cancer, both isoforms of 15- LOX are down-regulated in the cancerous epithelium than in the normal tissue, while a high level of the enzymes 12 and 5-LOX are expressed in the breast cancer sample and tissue [28]. The results of other studies show that increasing the expression level of 12-LOX and decreasing the level of 15-LOX in breast cancer patients play a prognostic role [29]. Similarly, in non-Small Cell Lung Cancer (NSCLC), the expression of 15-LOX-2 increased in well-differentiated tumors [26]. It can be concluded that the role of LOX expression in cancer growth depends on the type of tissue and the type of isoform that is expressed. To find the role of each isoform in cancer progression, it is necessary to examine the role of LOX metabolism in various aspects of cancer development, including cell growth, cell invasion, angiogenesis, and metastasis. In this review, we present recent evidence and findings indicating the role of the 15-LOX pathway in breast cancer and we also discuss the significance and importance of 15-LOX enzyme isoforms and main products on breast cancer cell growth, death, metastasis, and invasion. Identifying the role of the 15-LOX pathway, as a critical pathway in the process of lipid metabolism, in breast cancer pathogenesis may enhance our understanding of the putative mechanisms underlying breast tumor onset and progression and also may open up promising solutions for more effective treatments of this cancer.

## How 15-LOX works in a breast normal and tumor cell and what is the 15-LOX putative mechanism of action?

**Fig. 1:**
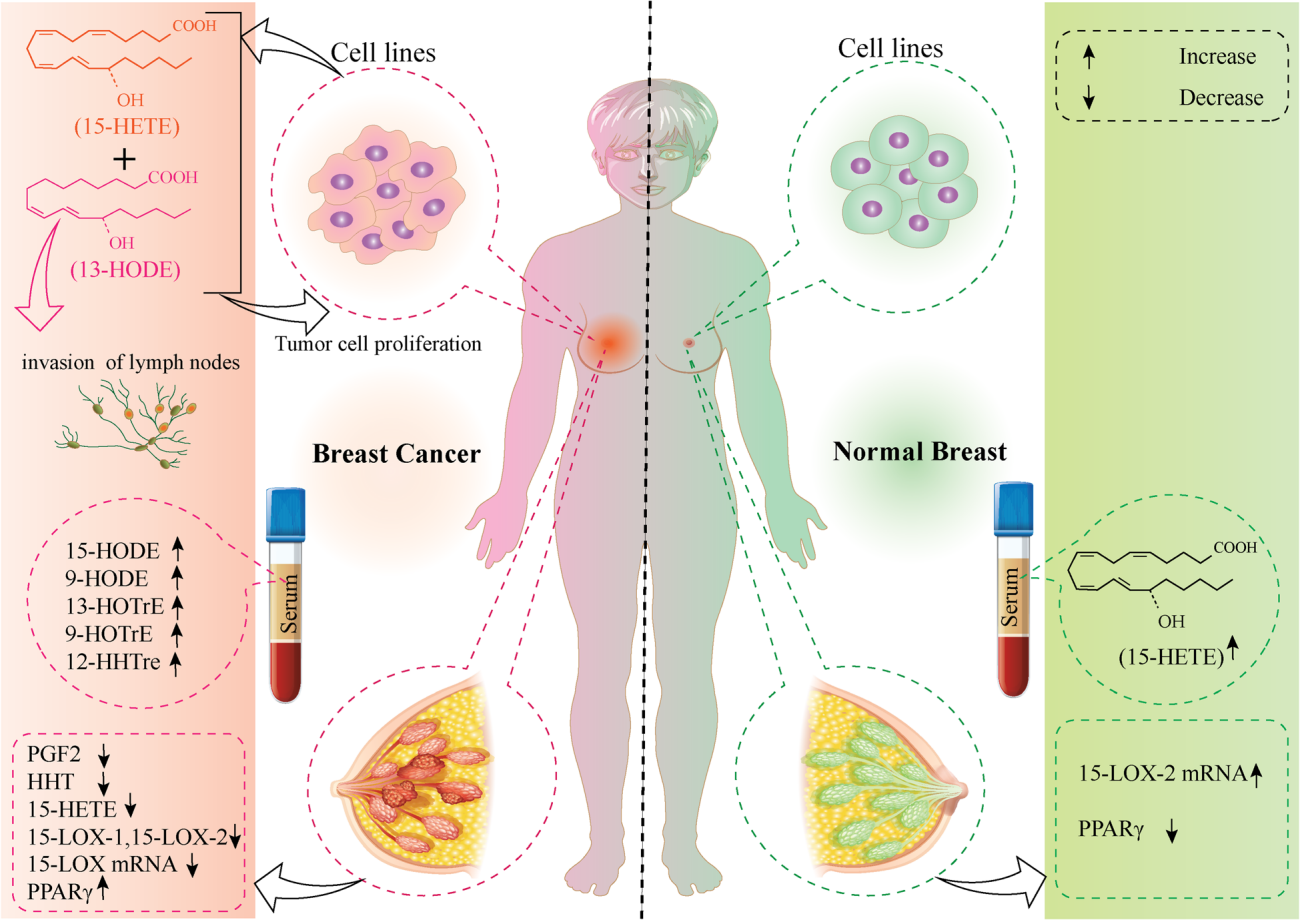
The expression pattern of 15-LOX and its related metabolites in breast tumor and normal tissues, the blood of patients and healthy subjects, and breast cancer cell lines. The local and circulating level of 15-LOX enzymes and their main metabolites is different in patients with breast cancer and healthy subjects. The 15-LOX and its metabolite levels are shown in healthy subjects (The bottom right of the image) and patients with breast cancer (The bottom left of the image). The expression level of the 15-LOX and the metabolites in the serum and tissue samples of individuals is shown separately. The decreasing level of 15-LOX-1, − 2 transcripts, and 15-HETE in breast tumor tissue was accompanied by the elevated level of PPARγ [17, 21, 25]. Besides the increasing level of 15-HODE, 9-HODE, 13-HOTrE, 9-HOTrE, and 12-HHTre was apparent in breast cancer patients versus the elevated level of 15-HETE in serum of healthy subjects [26, 36, 40]. 15-LOX activation through exogenous 15-HETE and 13-HODE administration leads to breast cancer cell proliferation and invasion that is illustrated in breast cancer cells (Top left of the image) and breast normal cells (top right of the image) [25, 40]

Given the breadth of 15-LOX-mediated cell death in breast tumors, the expression level of 15-LOX and its products have been surveyed in several studies that are summarized in Table 1. Evaluating the transcript level of 15-LOX in tumor tissues of patients with breast cancer showed a significant reduction of 15-LOX expression level compared to the normal breast tissues in node-positive patients [24]. The attenuated level of 15-LOX was associated with tumor stage and grade since the lowest level of 15-LOX was detected in TNM4 breast tumors. Also, the lower level of 15-LOX was detected in Lobular carcinomas compared to the ductal breast tumors [24]. Alongside with 15-LOX reduced level, over-expression of 12-LOX and cyclooxygenase-2 (COX-2) was observed in breast tumor tissues while 5-LOX level remained un-changed in breast tumor and normal tissues [28]. In accordance, it was shown that both 15-LOX isoforms (15-LOX-1 and 15-LOX-2) were expressed in breast normal epithelial cells and vascular endothelial cells while breast tumor cells showed lower protein levels of both 15-LOX isoforms compared to the normal breast cells. Also, in node-positive tumors, the 15-LOX-1 transcript was lower compared to the node-negative tumors. In addition, the lower level of 15-LOX-2: Cytokeratin 19 (CK19) ratio was detected in TNM3 tumors compared to TNM1 tumors indicating the trend of high grades tumors to down-regulate 15-LOX isoforms. Interestingly, in estrogen receptor (ER)-positive breast tumors, the expression level of 15-LOX-2 was reduced in the corresponding cells while the 15-LOX-1 expression level remained unchanged compared to the ER-negative tumors. The association of metastasis occurrence and development, tumor recurrence, and patient survival with a lower level of 15-LOX-2 and 15-LOX-1 was detected in patients with breast cancer. Based on the findings, the 15LOX1:15LOX2 ratio has prognostic value in predicting the clinical outcome of patients with breast cancer [24]. A high level of the 15-LOX-2 transcript was detected in normal prostate, breast, bladder, and skin tissues, while the expression level of 15-LOX-2 reduced remarkably in tumors of the mentioned tissues [30, 35, 36]. It might be explained by the hypothesis that 15-LOX-2 is localized in Golgi-like structures inside the normal epithelial cells, however, the mentioned structures are not found in the transformed cells. It was revealed that the 15-LOX-2 product (15-S-HETE) level was elevated in normal cells while reduced in the correspondence tumor cells. The regulatory role of PPAR-γ was demonstrated in the study of Subbarayan et al.; that based on their report, stimulation of PPAR-γ expression caused down-regulation of 15-LOX-2 in breast and lung normal epithelial cells. Accordingly, over-expression of 15-LOX-2 or 15-S-HETE in tumor cells was accompanied by reduced PPAR-γ protein level [30]. In another survey, the preoperative serum level of prostaglandin E2 (PGE2) and 6-keto-Prostaglandin F1 Alpha (6-k-PGF_1α_) in patients with malignant breast tumors was significantly higher compared to the postoperative levels of PGE2 and 6-k-PGF_1α_ [31]. The eicosanoid profile of breast tumor tissues indicated that the eicosanoid metabolites except for prostaglandin F2α (PGF_2α)_, heptadecatrienoic acid (HHT), and 15-Hydroxyeicosatetraenoic acid (15-HETE) were higher compared to the benign breast tumor or mammary reduction. A correlation was observed between tumor value of LOX products such as (15-HETE) and 13,14-dihydro-l5-keto-prostaglandin (DHKPG) with tumor diameter and fibrosis score. As a result, the eicosanoid synthesis profile showed a different pattern in mammary tumors and normal tissues [31]. In an in-vitro study, it was observed that 13(S)-HODE suppressed the growth of MCF-7 and MDA-MB-231 breast cancer cell lines in a dose/time-dependent manner. Moreover, the increasing concentration of 13(S)-HODE was accompanied by the accumulation of cells in the Sub-G1 phase of the cell cycle and the induction of early apoptosis. It was shown that the expression level of PPAR-γ was attenuated following treatment of breast cancer cells with 13(S)-HODE [37]. To clarify the expression status of 15-LOX-2 and its relationship with PPAR-γ, various normal and tumor tissues from epithelial and non-epithelial sources were enrolled in the survey. The normal epithelial cells derived from prostate, breast, lung, bladder, and skin tissues, expressed a high level of 15-LOX-2 gene and protein while low level of PPAR-γ. In contrast, the malignant tumors and the transformed epithelial cells of the prostate (PC-3 and DU145), breast (MDA453), lung (Calu I), bladder (U-9 and U-14), skin (HaCaT), and pancreas (Mia PaCa-2 and ASPC-1) exhibited a low level of 15-LOX-2 and high level of PPAR-γ. However, other epithelial cells of the same tissues including prostate (LNCaP), lung (MSK-3), breast (MCF-7 and SK-BR-3), and skin (SCC-M7 and SCC-P9), expressed a low level of 15-LOX-2 gene and protein while the level of PPAR-γ was not as high as the other malignant epithelial cells [30]. Treatment of MCF-7 cells with arachidonic acid and 15-L-_(S)_- hydroperoxy eicosatetraenoic acid (15-L-(S)-HPETE) induced cell toxicity in a time and dose-dependent manner and the cytotoxic effect of 15-L-_(s)_-HPETE, as a 15-LOX product was more remarkable [38]. It was found that 15-LOX-1 suppressed tumor growth and metastasis development in transgenic mice with mammary gland carcinoma and/ also mice with Lewis lung carcinoma [39]. In addition to studies that reported the decreased expression of the 15-LOX and its products, significant studies have shown that the LOX enzyme expression and activity increase in patients with breast cancer/ breast cancer cells (summarized in Fig. 1). In accordance, in the survey to determine the expression pattern of arachidonic acid metabolizing enzymes in a panel of human epithelial cancer cell lines, it was determined that cyclooxygenase-1 (COX-1), 5-lipoxygenase (5-LOX), and 5-lipoxygenase activating protein (FLAP) demonstrated a universal expression in all cells while the expression level of COX-2, 12-lipoxygenase (12-LOX), and 15-LOX revealed to be variable based on the type of cell line. For instance, the 15-LOX transcript was expressed in all types of colon and lung cancer cell lines while among 7 types of breast cancer cell lines (MB231, H2380, SKBR3, T47D, ZR75, MCF-WT, and MCF7-adr), only SKBR3 cells were not able to express 15-LOX also the expression of 15-LOX was not detectable in PC-3 (prostate cancer cells). Furthermore, it was shown that dual inhibition of LOX/COX enzymes (using 5,8,11,14- eicosatetraenoic acid (ETYA) and general inhibition of LOX enzymes (using Nordihydroguaiaretic acid (NDGA)) caused strong growth inhibition in SKBR3(breast cancer cell line), ZR75 (breast cancer cell line), T47D (breast cancer cell line), and COLO205 (colon cancer cell line) cells. While aspirin (ASA) as a general COX inhibitor, revealed little growth inhibitory effect on the mentioned cell lines indicating that the LOX inhibitors, unlike the COX inhibitors, were more putative in suppressing the growth of epithelial cancer cells regardless of the expression status of each enzyme [32]. To investigate the status of oxylipins in plasma of 20 patients with breast cancer and the age-matched healthy subjects, it was shown that the significant elevation 13-Hydroxyoctadecadienoic acid (13-HODE), 9-Hydroxyoctadecadienoic acid (9-HODE), 13S-hydroxy-9Z,11E,15Z-octadecatrienoic acid (13-HOTrE), 9-hydroxy-10E,12Z,15Z-octadecatrienoic acid (9-HOTrE), and 12S-hydroxy-5Z,8E,10E-heptadecatrienoic acid (12-HHTrE) was detected in the plasma of the breast cancer patients indicating that the LOX pathway metabolites from linoleic acid and linolenic acid metabolism are mainly up-regulated in breast cancer [33]. The metabolites of normal and tumor breast tissue of 27 patients were quantified using mass spectrometry. In accordance, it was shown that 13-HODE, 15-HETE, 12-Hydroxyeicosatetraenoic acid (12-HETE), 5-Hydroxyeicosatetraenoic acid (5-HETE), 5-Oxo-eicosatetraenoic acid (5-oxo-ETE), prostaglandin D2 (PGD2), and PGE2 metabolites were expressed in malignant and most of the normal breast tissues, while the only metabolite that its level was correlated with Mib1 scores, aggressive grade, and lymph node metastasis was 13-HODE. In contrast, a negative correlation was observed for PGE2 and PGD2 and aggressive features of breast tumors. To further determine the exact role of 13-HODE and 15-HETE on breast cancer cell growth, cells were exposed to different concentrations of these metabolites and it was shown that MDA-MB-231 and MCF-7 breast cancer cell number and proliferation was increased while PGD2 and PGE2 induced anti-proliferative effects in breast cancer cells. Also, no correlation was detected between the 13-HODE level and those of 15-HETE, 12-HETE, PGE2, or PGD2 in normal breast tissue, while the 13-HODE level was highly correlated with the 15-HETE level in tumors tissues of patients with 20 Mib1 scores [41]. To clarify the issue, it is worth mentioning that Mib1 is a cell proliferation marker that accounts for the tumor grading system based on summing the tumor differentiation, tumor necrosis, and the Mib1 score [42]. Taken together, activation of 15-LOX-1 and elevation of 13-HODE can stimulate breast cancer proliferation and invasion that possibly might shorten a patient’s survival rate [41]. The controversy was observed regarding the expression pattern of 15-LOX level in patients with breast cancer since the mRNA expression of 15-LOX was detected in breast cancer tumor tissues, however, the 15-LOX mRNA level was increased in tumor tissues of some patients and decreased in tumor tissues of other patients compared to the normal breast tissues [43]. Therefore, as with other aspects of tumor growth, the role of each 15-LOX isoform concerning breast cancer growth must be considered independently, given the origin of the tumor and the specific factors produced by the enzymes.

**Table 1:** The expression pattern of 15-LOX and its metabolites in breast cancer

Type of 15-LOX enzyme/ metabolites/ other mediators	Transcript/ protein level	Cell line/Human tissue type/serum	Observations	Ref
15-LOX	mRNA	Tumor breast tissue	• Reduction of 15-LOX level in tumor tissue • Association of lower expression level with tumor stage • Lower level of 15-LOX in Lobular carcinomas	[28]
15-LOX-1 15-LOX-2	mRNA Protein	breast normal epithelial cells/breast tumor tissues/ vascular endothelial cells	• Tumor tissues showed lower protein level of both 15-LOX isoforms compared to breast normal/ vascular endothelial cells • Association of 15-LOX isoforms down regulation with tumor severity, recurrence, metastasis and patient’s survival • Reduction of 15-LOX-2 and no change in 15-LOX-1 in ER-positive breast tumors	[24]
15-LOX-2 15-S-HETE	mRNA	Tumor/normal breast tissue	• High level of 15-LOX-2 transcript in normal vs tumor breast tissue • High level of 15-S-HETE in normal cells vs tumor cells • PPAR-γ mediates down-regulation of 15-LOX-2	[30]
Eicosanoid metabolites 15-S-HETE	Final product	Breast tumor tissues	• PGF_2α_, HHT and 15HETE were lower in tumors vs compared to the benign breast tumor or mammary reduction • Correlation between tumor value of 15-HETE with tumor diameter and fibrosis score	[31]
15-LOX-2 PPAR-γ	mRNA Protein	Normal epithelial cells/ malignant breast tumor tissue	• Normal epithelial cells expressed high level of 15-LOX-2 gene and protein while low level of PPAR-γ • Malignant breast tumors/ breast (MDA453) cells low level of 15-LOX-2 and high level of PPAR-γ • breast (MCF-7 and SK-BR-3) expressed low level of 15-LOX-2 gene and protein while the level of PPAR-γ was not as high as the other malignant epithelial cells	[30]
15-LOX	mRNA	MB231, H2380, SKBR3, T47D, ZR75, MCF-WT MCF7-adr	• SKBR3 cells was not able to express 15-LOX, while the rest of breast cancer cells expressed 15-LOX. • Dual inhibition of LOX/COX enzymes and general inhibition of LOX enzymes caused growth inhibition in SKBR3, ZR75,T47D cells.	[32]
13-HODE, 9-HODE 13-HOTrE 9-HOTrE 12-HHTrE	Final product	Plasma of patients with breast cancer	• 13-HODE, 9-HODE, 13-HOTrE, 9-HOTrE, and 12-HHTrE were elevated in the plasma of the breast cancer patients	[33]
13-HODE 15-HETE, 12-HETE 5-HETE 5-oxo-ETE PGD2 PGE2	Final product	Breast tumor tissues/ breast normal tissues/ MDA-MB-231/ MCF-7	• 13-HODE, 15-HETE, 12-HETE, 5-HETE, 5-oxo-ETE, PGD2, and PGE2 metabolites were expressed in malignant and most of normal breast tissues. • 13-HODE expression was correlated with aggressive grade and lymph node metastasis. • 13-HODE and 15-HETE induced proliferation of MDA-MB-231 and MCF-7.	[34]

## What is the role of 15-LOX products on breast normal cell proliferation and breast tumor cell growth, metastasis, and drug resistance?

**Fig. 2:**
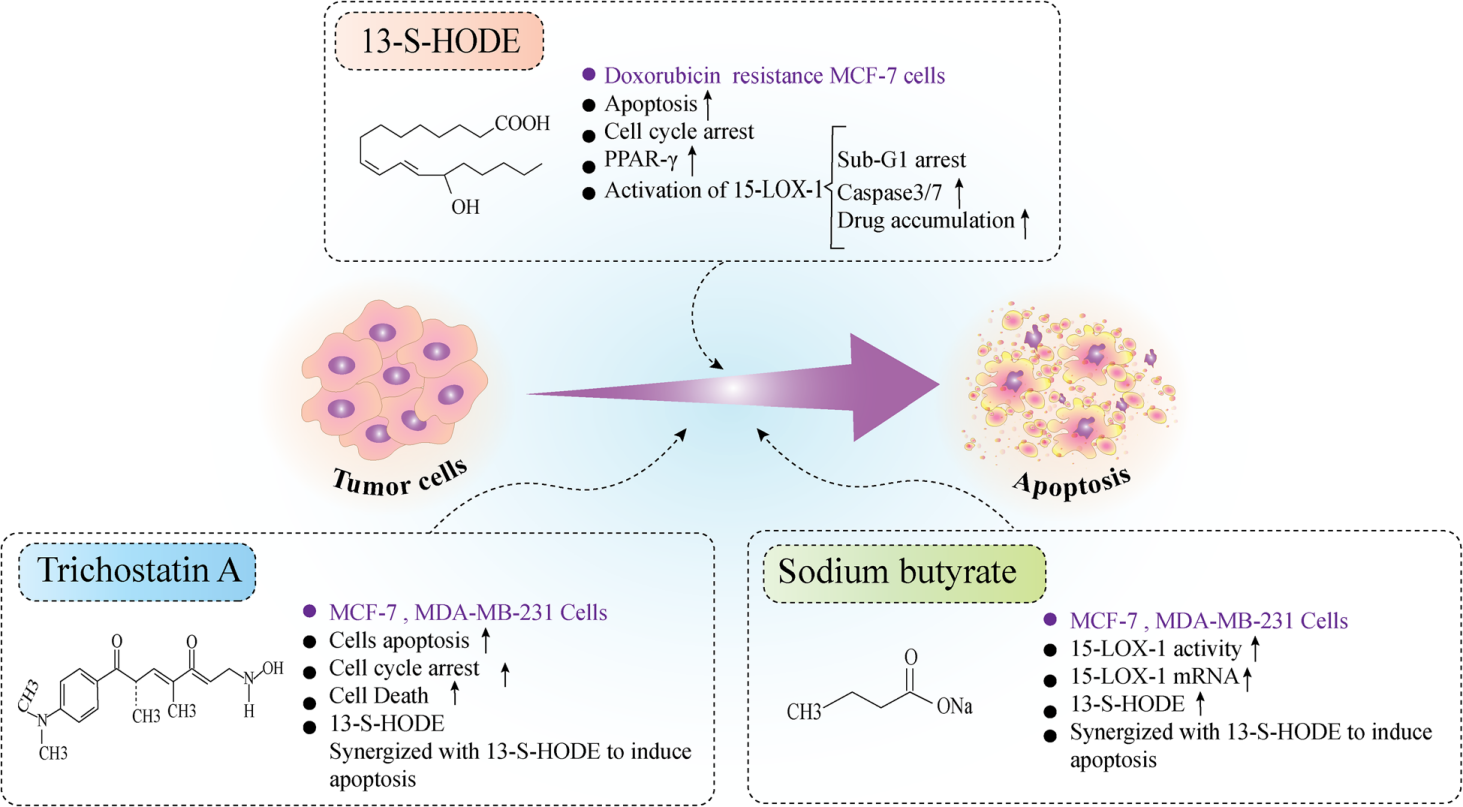
The pro-apoptotic effects of 15-LOX 1 in breast cancer cells. 15-LOX may mediate the regulation of breast cancer cell death by inducing apoptosis. Activation of 15-LOX and exogenous 13-HODE administration induce apoptosis and cell cycle arrest in doxorubicin-resistant MCF-7 cells [32]. Also, 15-LOX-1 mediates pro-apoptotic effects of Trichostatin A in MCF-7 and MDA-MB-231 cells possibly through elevation of 13-HODE [9]. sodium butyrate induced apoptosis in breast cancer cells through activation of 15-LOX-1 activity and transcript level [43]

Tumor cell growth is not only dependent on increased cell proliferation but also decreased cell death or apoptosis [44]. In cancer cells, the onset of apoptosis depends on the imbalance between pro-apoptosis and anti-apoptosis proteins and the downstream signaling pathways, which often involve caspase or mitochondrial cascades [45]. Evidence from previous studies suggests that 15-LOX may be effective in inducing and promoting cell death in tumors. In accordance, it was shown that in a doxorubicin-resistant breast cell line, 15-LOX-1 was down-regulated remarkably at both mRNA and protein levels compared to the doxorubicin-sensitive cells. Interestingly, the level of 13(S)-HODE showed to be lower in doxorubicin-resistant cells [46]. Based on the results it seems that following acquired resistance to doxorubicin, the 15-LOX-1 mRNA became less stable and no change in a copy number of 15-LOX-1 has occurred. Moreover, inhibition of 15-LOX-1 by (pSUPER-shALOX) shRNA vector in drug-sensitive parental cells were not able to induce doxorubicin-resistant phenotype indicating that other signaling pathways besides 15-LOX-1 activity might be involved in the induction of drug resistance phenotype. Although 15-LOX-1 over-expression enhanced drug accumulation, cell motility, subG1 arrest and apoptosis induction and, caspase 3/7 increased activity in doxorubicin-resistant MCF-7 cells, the aforementioned results were not obtained following 15-LOX-1 over-expression in HeLa doxorubicin-resistant cells [46]. It seems that 15-LOX-1 can interfere with the cell fate and induce apoptosis in a cell-dependent manner. Interestingly, exogenous 13(S)-HODE induced apoptosis and cell cycle arrest in doxorubicin-resistant MCF-7 cells that might be due to the activation of PPAR-γ. The lack of PPAR-γ expression in HeLa doxorubicin-resistant cells might be involved in a poor response of these cells to the cytotoxic effect of 13(S)-HODE [46]. Tavakoli-Yaraki et al.; reported that Trichostatin A (TSA) induced cell death and apoptosis in MCF-7 and MDA-MB-231 cells which were accompanied by the induction of apoptosis, cell cycle arrest, and increase in the level of 13(S)-HODE. However, 15-LOX-1 inhibition using PD146176 as a specific, non-competitive 15-LOX inhibitor, attenuated the pro-apoptotic effect of TSA in breast cancer cells also it was shown that 13(S)-HODE synergized with TSA to inhibit breast cancer cell growth and apoptosis. Therefore, based on evidence, it is indicated that the pro-apoptotic effect of TSA on breast cancer cell growth might occur through activation of 15-LOX-1 and its metabolite [16]. Most studies on the molecular mechanisms underlying 15-LOX-1-induced apoptosis have been implemented on colorectal cancer and the results show that 15-LOX-1 expression level is decreased in patients with colorectal cancer [25]. Also, the results of experiments on colorectal tumor cells show that the promoter of the 15-LOX-1 gene is under the strict control of several processes, including epigenetic processes [47, 48]. The potential regulatory role of epigenetic processes in controlling 15-LOX expression in breast cancer as well as colorectal cancer has been investigated. In accordance, it was shown that sodium butyrate as a short-chain fatty acid that is suggested to have histone deacetylase inhibitory effects, can regulate the growth of some types of cancer cells such as breast and colon [49]. Based on the evidence provided by Tavakoli-Yaraki et al.; sodium butyrate stimulated the activity of 15-LOX-1 through elevation of 13(S)-HODE level and 15-LOX-1 transcript. Also, it was reported that the rate of apoptosis and the percentages of apoptotic cells induced by sodium butyrate in MCF-7 and MDA-MB-468 cells was abrogated following pretreatment of the cells by PD146176. The simultaneous exposure of MCF-7 and MDA-MB-468 cells to the 13(S)-HODE and sodium butyrate was more effective in the induction of breast cancer cell death [50]. Table 2 and Fig. 2 summarize the involvement of 15-LOX in breast cancer cell growth …

**Table 2:** The anti-proliferative effects of 15-LOXand its metabolites in breast cancer cells

Type of 15-LOX enzyme/ metabolites/ other mediators	Cell line	Observations	Ref
15-LOX 13(S)-HODE	doxorubicin-resistant/ sensitive breast cell line	• 15-LOX-1 was down-regulated in doxorubicin-resistant cells vs sensitive cells. • level of 13(S)-HODE showed to be lower in doxorubicin-resistant cells. • 15-LOX-1 over-expression enhanced drug accumulation, cell motility, subG1 arrest and apoptosis induction, caspase 3/7 increased activity in doxorubicin-resistant MCF-7 cells. • exogenous 13(S)-HODE was enough to induce apoptosis and cell cycle arrest in doxorubicin-resistant MCF-7 through activation of PPAR-γ.	[46]
Trichostatin A 13(S)-HODE 15-LOX-1 PD146176	MCF-7 and MDA-MB-231	• Trichostatin A (TSA) induced cell death, apoptosis, cell cycle arrest and 13(S)-HODE elevation • 15-LOX-1 suppression diminished the pro-apoptotic effect of TSA in the breast cancer cells • 13(S)-HODE synergized with TSA to inhibit breast cancer cell growth and apoptosis	[16]
Sodium butyrate (SB)	MCF-7 and MDA-MB-231	• The activity of 15-LOX-1 and production of 13(S)-HODE level and15-LOX-1 transcript was stimulated by SB. • Inhibition of 15-LOX-1 eliminated the apoptosis induced by SB in the cells. • 13(S)-HODE synergized with SB to induce apoptosis in breast cancer cells.	[50]

## What is the interaction between COX and LOX enzymes with particular reference to 15-LOX?

**Fig. 3:**
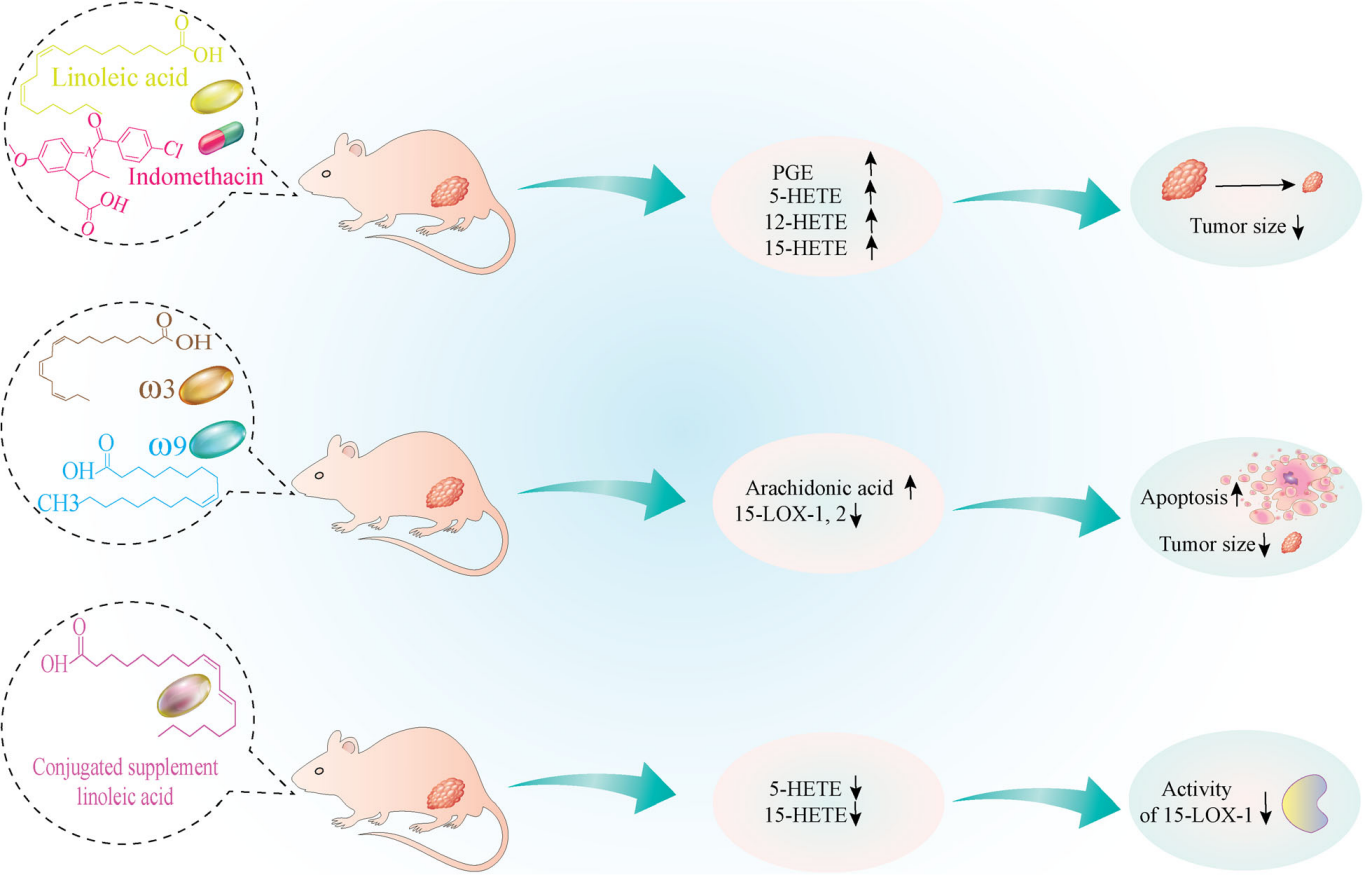
The effect of Linoleic acid-rich diet on 15-LOX activity and breast tumor behavior (In-vivo evidence). Changes in the activity of enzymes involved in fat metabolism can affect the fate of tumor cells. LA-enriched diet supplemented with indomethacin-induced 15-LOX metabolites and reduced breast tumor size in nude mice [45]. The peanut oil-enriched diet enriched by ω-3 and ω9 PUFAs caused elevation of membrane arachidonic acid (AA) content and reduced activity of 15-LOX-2 and 15-LOX-1 that was accompanied by increased apoptosis and decrease mitosis in murine mammary gland adenocarcinoma [52]. The conjugated linoleic acid (CLA) in pregnant and breastfeeding female rats was associated with reduced LOX metabolites and enzyme activity lower risk of mammary tumor size induced by chemicals in their offspring [51, 53]

**Fig. 4:**
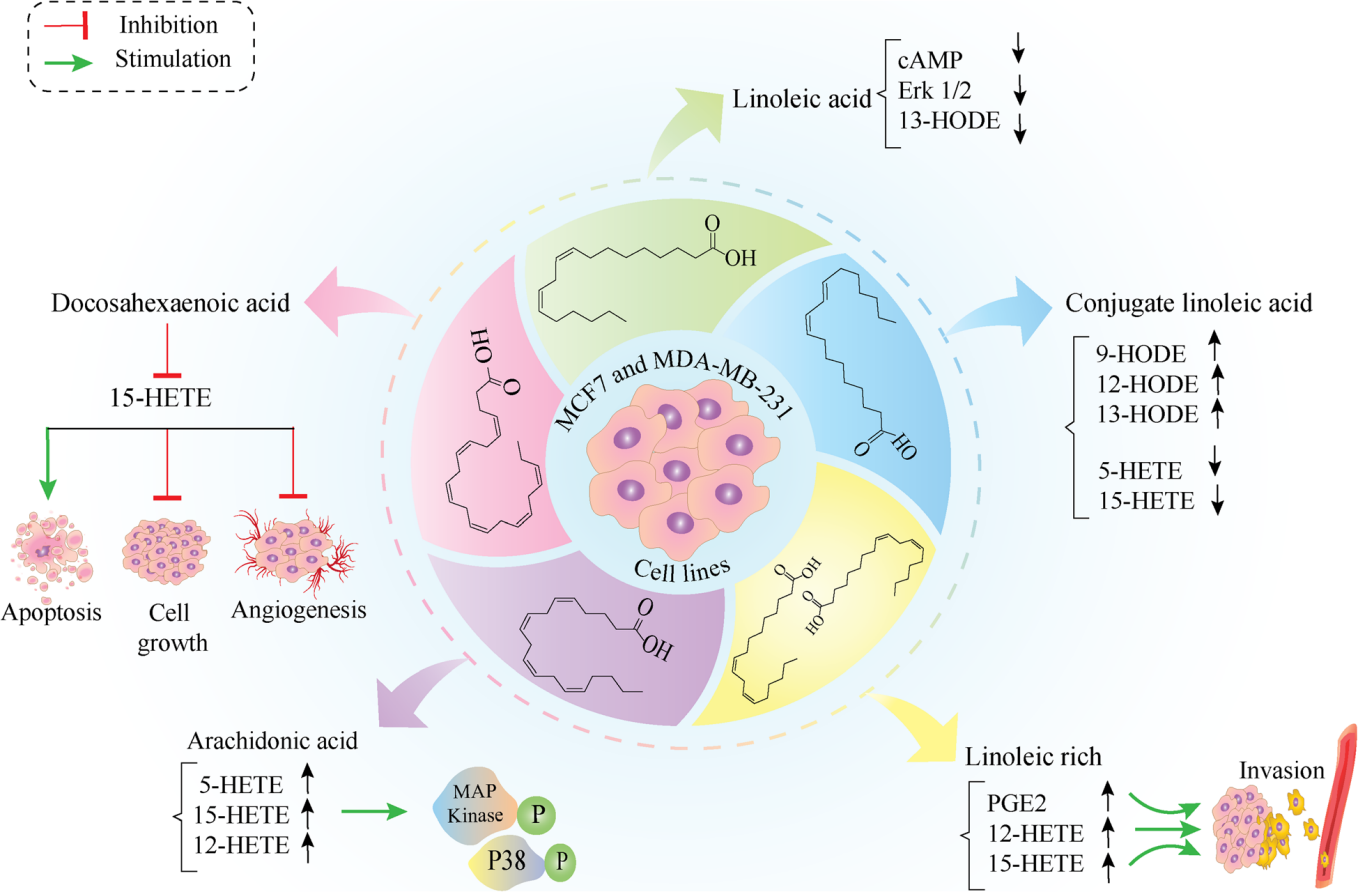
The effect of exogenous lipid metabolism modulation on breast cancer cell behavior and 15-LOX metabolites (In-vitro evidence). Various compounds that target the metabolism of fatty acids could be effective in the growth and invasion of breast tumor cells through lipoxygenase metabolites. Docosahexaenoic acid reduced tumor cell growth, cell proliferation, tumor-induced angiogenesis through inhibition of 15-HETE synthesis [47]. The cAMP content, activity of Erk1/2 and 13-HODE synthesis were suppressed following linoleic acid isomers administration [48]. The conjugated linoleic acid (CLA) reduced the level of 15-HETE and 5-HETE while exogenous arachidonic acid stimulated production of 15(*S*)-HETE and phosphorylation of p38 MAPK [54]. The linoleic acid-rich supplement stimulates the production of PGE2, 12-HETE, and 15-HETE which was associated with tumor cell invasion [46]

As it is well established, improper regulation of the balance between cell proliferation and cell death results in tumor formation and is a hallmark of cancer [44]. Many cellular pathways interfere with the process of cell stagnation, apoptosis, or cell proliferation and it was revealed that LOX products cooperate with different growth factor signaling cascades to stimulate the growth of tumor cells [51]. It was reported that in nude mice under a high-fat diet enriched with linoleic acid, administration of indomethacin (A cyclooxygenase inhibitor) resulted in a higher concentration of prostaglandin E (PGE), 5-HETE, 12-HETE, and 15-HETE, which in tumors from the control group under high-fat diet enriched with linoleic acid (LA-enriched diet) than in those under diet contains low linoleic acid that was associated with reducing the risk of breast cancer progression in nude mice fed by LA-enriched diet (summarized in Fig. 3) [53]. In support of this evidence, it was shown that the LA-enriched diet can stimulate the MDA-MB-435 cells to produce COX and LOX products such as PGE2, 12-HETE, and 15-HETE which was associated with cell invasion. While, inhibition of 12-LOX, but not COX, can abolish eicosanoid metabolites secretion and cell invasion also stimulate the activity of metalloproteinase-9. It can be postulated that the effect of an LA-enriched diet on breast cancer cell growth can be at least partly due to the role of the lipoxygenase enzymes specially 12-LOX [54]. In addition to studies evaluating the role of 15-LOX in the animal model, several studies have examined the above mechanisms at the cellular level, which are demonstrated in Fig. 4. It was shown that inhibition of 15-HETE synthesis using docosahexaenoic acid administration was associated with reduced tumor cell growth and cell proliferation, increased apoptotic cells, and inhibition of tumor-induced angiogenesis [55] (Fig. 4). Also, it was shown that LA isomers suppress the uptake of linoleic acid, the content of cyclic adenosine monophosphate (cAMP) and the activity of extracellular signal-regulated protein kinase 1/2 (Erk1/2), and production of 13-HODE in steroid receptor-negative (SR2) MCF-7 cells possibly in a receptor-mediated manner. In addition, a small amount of 15(S)-HETE was produced in tumor tissue of MCF-7 (SR2) human breast xenografts. The combination of 13-HODE with linoleic acid isomers resulted in the elevation of cAMP level and emphasized the impact of 13-HODE on cAMP level (through activation of adenylate cyclase or suppression of phosphodiesterase) at a pharmacological level however the underlying mechanism needs to be clarified [56] (Fig. 4). It was shown that treatment of MDA-MB-231 cells with conjugated linoleic acid (CLA) (t10, c12-CLA), reduced the level of 15-HETE and 5-HETE. Also, incubation of proteins that were extracted from MDA-MB-231 cells with arachidonic acid, resulted in the production of 15- HETE, 12- HETE, and 5-HETE while simultaneous incubation of proteins with arachidonic acid and c9, t11-CLA or t10, c12-CLA did not affect the level of metabolite production. So, it can postulate that 5-, 12- and 15-LOX-2 enzymes have minimum effect on metabolizing c9, t11-CLA or t10, c12-CLA in breast cancer. Also, based on the evidence of this study 15-LOX-1 enzyme was not able to metabolize the aforementioned isomers while the linoleic acid is metabolized by 15-LOX-1 and produces 13-HODE [52]. In a study to investigate the impact of CLA supplements on arachidonic acid metabolites (15-, 12-, 5-HETE) and linoleic acid (13-, 9-HODE) levels during induction of breast tumors in rats, it was shown that the 5-HETE, 9-HODE, 12-HETE and 13-HODE levels were enhanced while the highest level belonged to the 5-HETE and 15-HETE in 7,12-dimethylbenz [a] anthracene (DMBA)-induced tumor group. While the 13-HODE level was dominated in the oil-DMBA-induced tumor group, however; its level was lower in the group treated with CLA- DMBA [57, 58]. It was shown that a high CLA diet in pregnant and breastfeeding female rats was associated with a lower risk of mammary tumor induced by chemicals in their offspring. It seems that the concentration of LOX metabolites was effective in this regard. Receiving the CLA supplements was associated with a higher concentration of 15-HETE in serum indicating that the LOX metabolites of arachidonic acid (15-, 12-, 5-HETE) and linoleic acid (HODE) were higher in mammary tumors in groups under the CLA diet. In accordance, there is a competition between CLA and PUFAs that might affect the PUFAs and their LOX metabolites concentrations [59]. It was shown that treatment of MDA-MB-435 breast cancer cells with exogenous arachidonic acid resulted in the production of 15(*S*)-HETE. While in the presence of NDGA (a LOX inhibitor), the exogenous arachidonic acid failed to produce 15(S)-HETE, and the phosphorylation of p38 and mitogen-activated protein kinase (MAPK) was inhibited (Fig. 4). Interestingly the protein level of 15-LOX-2 but not 15-LOX-1 was increased in MDA-MB-435 cells suggesting that 15-LOX-2 was responsible to metabolize exogenous arachidonic acid to 15(*S*)-HETE. Also, treatment of MDA-MB-435 cells with exogenous 15(*S*)-HETE resulted in an activation of p38 and MAPK pathway and cell adhesion to type IV collagen indicating the pushing role of 15(*S*)-HETE produced by 15-LOX-2 in activation of signaling pathways related to the cell adhesion to the extracellular matrix [60]. Interestingly it was shown that the circulating level of eicosapentaenoic acid (EPA) inhibits the proliferation of human MCF-7 xenografts that might be mediated by reduction of linoleic acid uptake and 13-HODE formation. The lower level of 13-HODE was accompanied by a decrease in cAMP and ERK1/2 phosphorylation in MCF-7 xenografts. In the presence of exogenous 13-HODE to the EPA-containing arterial blood, the uptake of 13-HODE by tumors enhanced, the tumor DNA content increased, and the phosphorylated ERK1/2 was restored [61]. In a study on MCF-7 human breast xenografts, a perfusion system was designed and the tumors were perfused with rat blood that was supplemented with melatonin (MLT), EPA, and CLA promptly and the tumor uptake of linoleic acid and its conversion to 13-HODE and the subsequent release to the blood circulation were monitored. It was revealed that the linoleic acid uptake and 13-HODE generation was suppressed and the phosphorylation of ERK1/2 was significantly reduced following receiving anticancer agents suggesting the association of 13-HODE release rate and ERK1/2 phosphorylation [62]. Also, it was revealed that the peanut oil-enriched diet which is rich in ω-3 and ω9 PUFAs caused elevation of membrane arachidonic acid content and reduced activity of 15-LOX-2 and 15-LOX-1 that was accompanied by increased apoptosis and decrease mitosis in murine mammary gland adenocarcinoma (Fig. 3). The reduced tumor volume, number of metastasis, and longer survival were detected in the mice group under a peanut oil-enriched diet. In support of this, it was proposed that the reduced severe features of tumors were related to the suppression of LOXs activity and decrease of pro-tumorigenic eicosanoids such as 15 (S)-HETE [63]. Tables 3 and 4 summarize the tumorigenic effect of 15-LOX and its products in breast cancer tissue and cells.

**Table 3:** The involvement of 15-LOX and its metabolites in the pro-tumorigenic effects of various conjugated lipid-based diets in breast cancer; in vivo evidences

Type of 15-LOX enzyme/ metabolites examined	Cell line/Human tissue type/serum	Type of intervention	Observations	Ref
PGE, 5-HETE 12-HETE 15-HETE	Nude mice tumor tissue	LA-enriched diet	• Indomethacin caused elevation in the level of PGE, 5-HETE, 12-HETE, and 15-HETE in tumors of the group under LA-enriched diet Indomethacin might reduce the risk of breast cancer progression in nude mice fed by LA-rich diet.	[53]
LA-derived prostaglandin E2 12-HETE 15-HETE	MDA-MB-231 cell solid tumor	Docosahexaenoic acid diet containing 4% linoleic acid	• Inhibition of 15-HETE synthesis using docosahexaenoic acid administration caused reduced breast tumor cell growth, suppression of cell proliferation, increased apoptotic cells and inhibition of tumor-induced angiogenesis	[55]
5-HETE 9-HODE 12-HETE 13-HODE	Rat breast tumors	CLA supplements	• CLA supplements induced production of 5-HETE, 9-HODE, 12-HETE and 13-HODE. • 5-HETE and 15-HETE in 7,12-dimethylbenz [a] anthracene (DMBA)-induced tumor group. • CLA- DMBA treated group induced low level of 13-HODE compared to oil-DMBA-treated group.	[58]
LOX metabolites	Pregnant and breastfeeding female rats	CLA diet	• LOX metabolites of arachidonic acid (15-, 12-, 5-HETE) and linoleic acid (HODE) were higher in mammary tumors in groups under CLA diet.	[59]
13-HODE	MCF-7 xenografts	Eicosapentaenoic acid (EPA)	• EPA inhibit proliferation of human MCF-7 xenografts through linoleic acid uptake and 13-HODE formation. • The lower level of 13-HODE was accompanied with decrease in cAMP and ERK1/2 phosphorylation. • Exogenous 13-HODE caused uptake of 13-HODE by tumors, increased the tumor DNA content, restored the phosphorylated ERK1/2.	[59]
LA 13-HODE	MCF-7 human breast xenografts	melatonin (MLT), eicosapentaenoic acid (EPA), conjugated linoleic acid (CLA)	• Receiving anticancer agents (MLT, EPA, CLA) resulted in suppression of LA uptake, 13-HODE generation and phosphorylation of ERK1/2.	[62]
Arachidonic acid (AA) content 15-LOX-1 15-LOX-2	Murine mammary gland adenocarcinoma.	Peanut oil enriched diet rich in ω-3 and ω9 PUFAs	• Peanut oil enriched diet which is rich in ω-3 and ω9 PUFAs caused elevation of membrane AA content, reduced 15-LOX-2 and 15-LOX-1 activity, increased apoptosis, decrease mitosis. • Peanut oil enriched diet reduced tumor volume, number of metastasis and longer survival of rats.	[63]

**Table 4:** The involvement of 15-LOX and its metabolites in the pro-tumorigenic effects of various conjugated lipid-based diets in breast cancer; in vitro evidences

Type of 15-LOX enzyme/ metabolites examined	Cell line/Human tissue type/serum	Type of intervention	Observations	Ref
PGE2 12-HETE 15-HETE	MDA-MB-435 cells	LA-enriched supplement	• linoleic acid-rich supplement stimulate production of PGE2, 12-HETE and 15-HETE which was associated with cell invasion. • Inhibition of 12-LOX abolish eicosanoid metabolites secretion, cell invasion and activate metalloproteinase-9	[54]
13-HODE 15(S)-HETE	Steroid receptor negative (SR2) MCF-7	Linoleic acid isomers/13-HODE	• Uptake of linoleic acid, the content of cAMP and the activity of Erk1/2 and production of 13-HODE were inhibited following linoleic acid isomers administration. • linoleic acid isomers caused production of 15(S)-HETE in tumor tissue of MCF-7 (SR2) human breast xenografts. • 13-HODE synergize with linoleic acid to elevate cAMP level.	[56]
15-HETE 5-HETE 12- HETE	MDA-MB-231	Conjugated linoleic acid (CLA)	• CLA (t10, c12-CLA) reduced 15-HETE and 5-HETE level. • Arachidonic acid caused production of 15- HETE, 12- HETE and 5-HETE while incubated with cell extracted proteins. • 5-, 12- and 15-LOX-2 have minimum effect on metabolizing c9, t11-CLA or t10, c12-CLA in breast cancer. • 15-LOX-1 was not able to metabolize c9, t11-CLA or t10, c12-CLA isomers.	[52]
15(*S*)-HETE 15-LOX-1 15-LOX-2	MDA-MB-435	Exogenous arachidonic acid/ exogenous 15(*S*)-HETE	• Exogenous arachidonic acid results in the production of 15(*S*)-HETE and phosphorylation of p38 MAPK that were abrogated following LOX inhibition. • Exogenous arachidonic acid induced protein expression of 15-LOX-2 but not 15-LOX-1. • Exogenous 15(*S*)-HETE activate p38 MAPK pathway and cell adhesion to type IV collagen	[60]

## How anti-cancer drugs act on 15-LOX

Although it may seem a bit off-topic, interesting studies have been found on the relationship between melatonin, and 13-HODE in breast cancer. It was shown that in human MCF-7 breast cancer xenografts, exposure to constant light stimulates tumor tissue growth. Interestingly, the LA uptake and 13-HODE production in the group that was constantly exposed to the light was increased compared to the control group. The constant light exposure-induced tumor growth can be explained by the suppression of melatonin (MLT) synthesis, increased linoleic acid uptake, and production of 13-HODE [64]. Based on multiple lines of evidence, neurohormone MLT suppresses several human tumor cell growth and metabolism. It seems that the anticancer effect of melatonin occurs in a receptor-mediated manner through inhibition of linoleic acid uptake by tumor cells. It is well documented that linoleic acid is a fatty acid that promotes tumor growth and converts to the 13-HODE which has a mitogenic effect. The perfusion of MCF-7 human breast xenografts by melatonin in different time schedules resulted in suppression of linoleic acid uptake and 13-HODE generation by tumors and subsequently decrease the ERK1/2 phosphorylation [62]. Following inhibition of melatonin in dim light at night, the balance among various preventive signaling pathways in cancer is disrupted and resulted in hyperglycemia, hyperinsulinemia, and proliferative activity in the tumor. In addition, during exposure to the light the fatty acid, and linoleic acid uptake, 13-HODE formation, cAMP level, glucose uptake and lactate production increased that was abrogated when exposed to the dark. It was revealed that the control of tumor cell metabolism, aerobic glycolysis, and growth was dependent on the melatonin-induced inhibition of 13-HODE formation and subsequently the activation of protein kinase B (AKT) by 13-HODE. Interestingly, melatonin itself was able to down-regulate phospho-Akt (Ser473) (pAKTs473) and reduce the activity of AKT. The observed effects were diminished in the presence of MT1/MT2 melatonin receptor antagonist S20928. It was concluded that melatonin suppresses linoleic acid uptake, 13-HODE formation, and the Warburg effect in human breast cancer xenografts and 13-HODE play signaling like to activate Warburg effect by AKT activation [65].

## How interaction between breast tumor cell-normal cell-tumor microenvironment is influenced by 15-LOX? (The consequent impact on breast tumor cell growth)

**Fig. 5:**
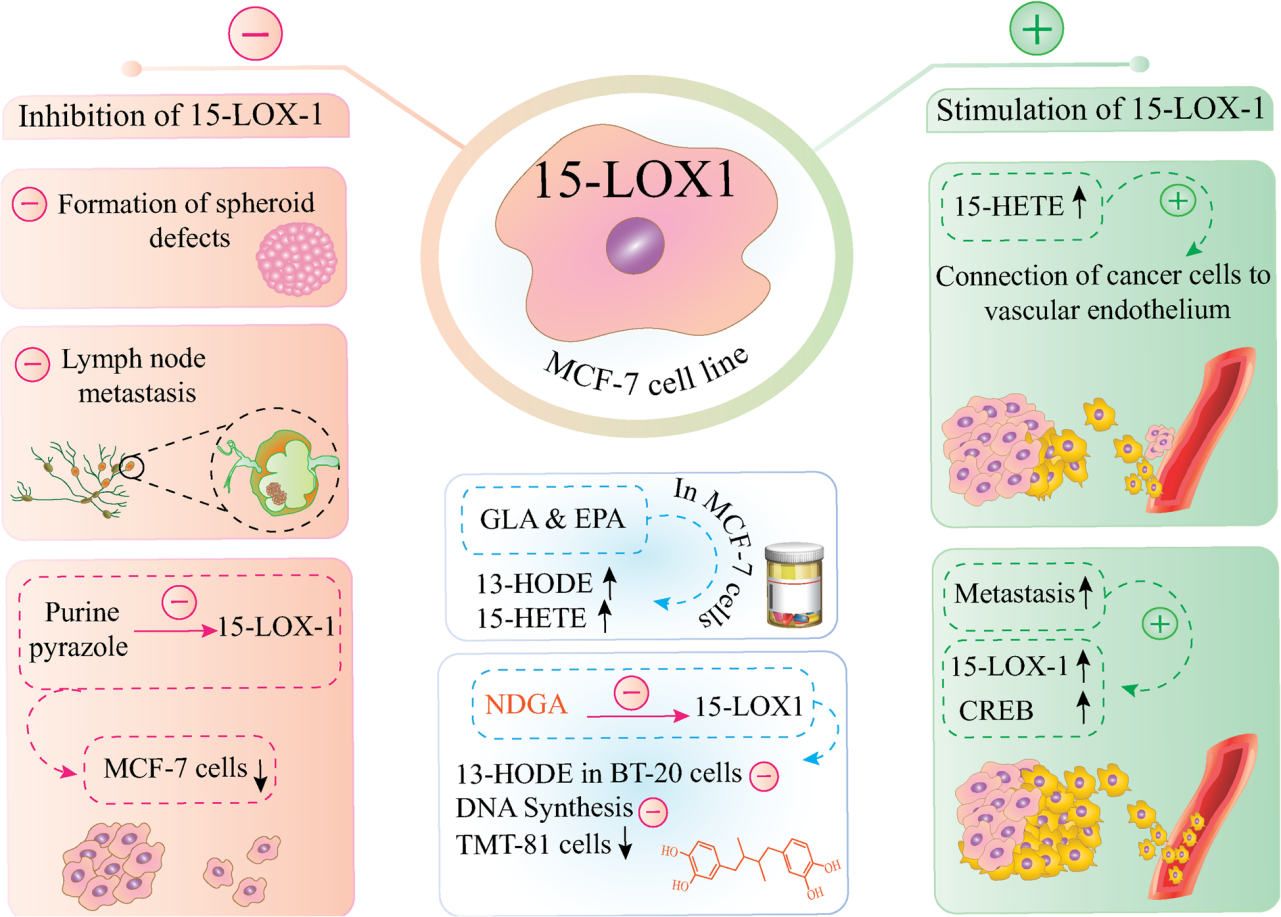
The effect of 15-LOX and its mediators on breast cancer cell metastasis and invasion. The 15-LOX activity can affect breast cancer cell metastasis and invasion through various pathways. Inhibition of 15-LOX activity leads to reduced MCF7 spheroid formation and metastasis to the lymph node [61]. The MCF-7 cell exposure to EPA and GLA induced the production of 15(S)-HETE and 13(S)-HODE [63]. 15-LOX-1accounts as a positive activator of CREB that mediates breast cancer cell metastasis [64]. 15-LOX-1 suppression using novel purine-pyrazole hybrids induced MCF-7 cell death, however; 15-LOX inhibition using NDGA caused TMT-081 cell growth suppression, reduced DNA synthesis, and 13-HODE formation [40, 64, 67]. The attachment of breast cancer cells to the lymph-endothelial cells facilitates using 12(S)-HETE generation by 15-LOX-1 [61, 62]

Studies of the last two decades have shown that there are several factors involved in the onset and progression of breast cancer [40]. A considerable number of breast cancer patients experience tumor metastasis and tumor recurrence that leads to premature death. Therefore, recognizing the factors involved in initiating tumor metastasis and the underlying mechanism is helpful in clinical decision-making [66]. Regarding the relevance of the 15-LOX pathway in breast cancer metastasis, it was revealed that the 15-LOX-1 gene induced in MCF-7 cell spheroids when compared with endothelial monolayers. Following enzyme activity inhibition using NDGA, the MCF-7 spheroid–induced circular defects in lymphatic endothelial cell monolayers were remarkably decreased in a dose-dependent manner (Fig. 5). In addition, knockdown of 15-LOX-1 activity using shRNA caused suppression of circular defects formation as well as metastasis to the lymph node in breast xenografted tumors. Based on this study, 15-LOX-1 is involved in mediating tumor cell invasion and lymph node metastasis in breast carcinoma [68]. In support of this, simultaneous inhibition of 15-LOX-1 using baicalein and nuclear factor kappa B (NF-κB) using Bay11–7082 (an inhibitor of κB kinase (IKK)) caused inhibition of circular chemorepellent-induced defects’ (CCID) formation in MCF-7 cell spheroids. While attaching to the lymph-endothelial cell monolayers, CCID is considered as a tumor intravasation process and it seems that generation of 12(S)-HETE by 15-LOX-1 under NF-κB regulation can facilitate CCID and attachment of breast cancer cells to the lymph-endothelial cells [67]. In addition, exposure of breast cancer cells to the EPA resulted in an increased expression level of E-cadherin, while treatment of the cells with gamma linoleic acid (GLA) did not affect E-cadherin level. However, both GLA or EPA treatments caused elevation of 15(S)-HETE and 13(S)-HODE level in MCF-7 cells, and a higher level of 13(S)-HODE was observed when compared to 15(S)-HETE although the reduced expression of E-cadherin in breast cells seemed to be dependent to the ratio of 15-LOX-1 metabolites [69]. The proteome study in myeloid-derived suppressor cells (MDSCs) in response to metastatic breast tumors, demonstrated that in MDSCs the selective expression of lipid metabolic pathway occurs. It was found that in MDSCs from metastatic 4 T1 tumors, the 15-LOX-1 was a positive activator of cAMP Response Element-Binding Protein (CREB) which is a transcription factor and is involved in the biological process in MDSCs [70]. In another study, it was shown that while bovine mammary endothelial cells (BMEC) were exposed to *Streptococcus uberis* no effect was detected on 15-LOX-1 metabolite levels, while exposure of bovine monocytes to *Streptococcus uberis* resulted in the production of 13-Hydroperoxyoctadeca-cis-9, trans-11-dienoic acid (13-HPODE) and 13-HODE during mastitis. Interestingly, treatment of BMEC with 13-HPODE reduced endothelial barrier integrity and accelerate apoptosis while co-treatment with antioxidants, reverses the previously seen effects [71]. It was found that 15-LOX-1 suppression using novel purine-pyrazole hybrids which contain thiazoles, thiazolidinones and rhodanines reduced the rate of viable MCF-7 cells and proposed to act as an anticancer compound in breast cancer [34, 72, 73]. It was shown that the exogenous arachidonic acid was metabolized in TMT-081 rat mammary tumor cell line and the range of eicosanoids metabolites including lipoxygenase and cyclooxygenase metabolites were produced. In accordance, it was observed that the DNA synthesis was stimulated in response to the 15-HETE and LOX inhibition using NDGA and esculetin that resulted in TMT-081 cell growth suppression [74]. It was shown that incubation of BT-20 cells (triple-negative breast cancer cells) with Transforming growth factor-alpha (TGF-α) and A23187, a mobile ion-carrier, caused 13-HODE formation which was dependent on the Epidermal growth factor (EGF) / TGF-α. While by suppression LOX activity, the TGF-α-dependent generation of 13-HODE and stimulation of DNA synthesis was abrogated. The findings indicated that LOX activity and 13-HODE production are involved in transferring the mitogenic signals that transferred from EGF/TGF-α in the cell surface to the cell nucleus in breast cancer [75]. The relation of 15-LOX and its products with breast cancer metastasis and invasion is summarized in Table 5.

**Table 5:** The effects of 15-LOX and its metabolites in breast cancer metastasis and invasion

Type of 15-LOX enzyme/ metabolites examined	Cell line/Human tissue type/serum	Type of treatment	Observations	Ref
15-LOX-1	MCF7/ endothelial monolayers	15-LOX-1 activation 15-LOX-1 inhibition	• 15-LOX-1 gene activation induced in MCF7 cell spheroids. • 15-LOX-1 inhibition/knock-down leads to MCF7 spheroid–induced circular defects in lymphatic endothelial cell monolayers decrease. • 15-LOX-1 knock-down suppress formation of circular defects and metastasis to the lymph node in breast xenograft tumors.	[68]
15-LOX-1 12(S)-HETE	MCF-7	15-LOX-1 inhibition	• 15-LOX-1 inhibition (baicalein) and NF-kB (Bay11–7082) caused inhibition of circular chemo repellent-induced defects’ (CCID) formation in MCF-7 cell spheroids. • 12(S)-HETE generation by 15-LOX-1 under NF-kB facilitate CCID and attachment of breast cancer cells to the lymph-endothelial cells.	[67]
15(S)-HETE 13(S)-HODE	MCF-7	Exposure to eicosapentaenoic (EPA), gamma linoleic acid (GLA)	• EPA treatment caused increased expression level of E-cadherin while GLA treatment had no effect. • GLA or EPA treatment caused elevation of 15(S)-HETE and 13(S)-HODE level	[69]
15-LOX-1	Myeloid derived suppressor cells (MDSCs), metastatic breast tumors		• In MDSCs from metastatic 4 T1 tumors, 15-LOX-1 was a positive activator of CREB.	[70]
13-HPODE 13-HODE	Bovine mammary endothelial cells (BMEC), bovine monocytes	*Streptococcus uberis*	• 15-LOX-1 metabolite levels remained unchanged in BMEC exposed to *Streptococcus uberis*. • 13-HPODE and 13-HODE produced in bovine monocytes exposed to *Streptococcus uberis*. • BMEC treatment with 13-HPODE reduced endothelial barrier integrity, accelerate apoptosis while the effects reversed in co-treatment with antioxidant.	[71]
15-LOX-1	MCF-7	purine-pyrazole hybrids	• 15-LOX-1 suppression using purine-pyrazole hybrids reduced the rate of viable MCF-7 cells	[34]
15-HETE	TMT-081 rat mammary tumor cell line	Exogenous arachidonic acid/ to 15-HETE	• 15-HETE stimulate DNA synthesis in TMT-081 cells. • LOX inhibition using NDGA and esculetin resulted in TMT-081 cell growth suppression. • Exogenous arachidonic acid induced production of eicosanoids metabolites in TMT-081.	[74]
13-HODE LOX	BT-20 cells	TGF alpha A23187	• TGF alpha and A23187 caused 13-HODE formation in BT-20 cells. • LOX activity suppression reduced 13-HODE formation and DNA synthesis. • LOX activity and 13-HODE production are involved in transferring the mitogenic signals in breast cancer cell.	[75]

## How are 15-LOX and oxidative stress connected to regulate breast cancer cell growth?

**Fig. 6:**
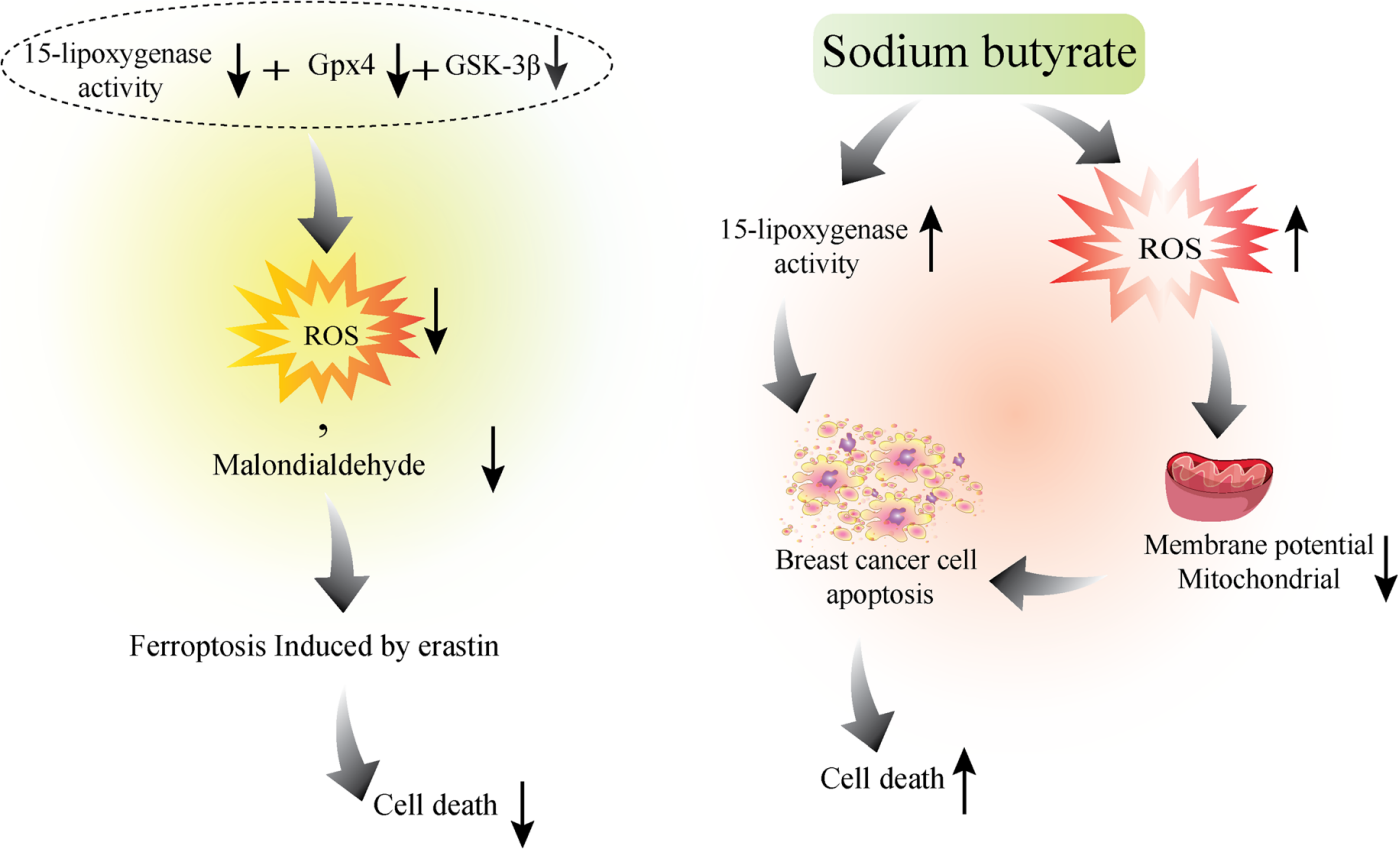
The relevance of 15-LOX and the oxidative stress in breast cancer cell growth. The reduction in the expression level of 15-LOX-1 is associated with a decrease in the level of ROS and reduced breast cancer cell death [74]. Also, 15-LOX-1 activation-induced breast cancer cell apoptosis might occur through the generation of ROS [75]

It is taken for granted that increased lipid peroxidation and oxidative stress account as crucial risk factors for breast cancer incidence, development, and progression [76]. On the other hand, tumor cell fate, growth, invasion and, death are influenced by lipid metabolism, peroxidation, and level of saturation [77]. As a consequence of aerobic metabolism, reactive oxygen species (ROS) are continually generated and transformed in the normal and tumor cells that are involved in various cell functions including cell proliferation and death. Therefore, maintaining redox balance by increasing antioxidant capacity and reducing the production of ROS is essential for the survival of tumor cells [78]. In support of this, the catalytic activity of LOX enzymes leads to the production of lipid peroxides that are involved in different cell signaling events regulating tumor cell growth or death. Notably, overproduction of lipid peroxides alongside iron accumulation can trigger ferroptosis which accounts for a type of programmed cell death [79]. It was shown that upon ROS stress, activation of 12-LOX induced ferroptosis in tumor cells through overproduction of lipid peroxides [7]. The regulatory role of 15-LOX in inducing the above cell death seems more important and interesting. It was shown that glutathione peroxidase 4 (GPX4) as a protective enzyme against lipid peroxidation as well as 15-LOX can induce nuclear factor erythroid-2 like 2 (NFE2L2) (Nrf2) as a regulator of antioxidant response in tumor cells that suppress ferroptosis and contributes to tumor cell chemoresistance [79]. Although the role of 15-LOX and its metabolites in stimulating various intracellular pathways leading to the regulation of oxidative stress and tumor cell fate can be extensive, the evidence in breast cancer is very limited. In the most relevant study, Xinghan Wu et all demonstrated that inhibition of Glycogen synthase kinase-3β (GSK-3β) resulted in decreased ROS and malondialdehyde (MDA) level through activation of GPX4 and inhibition of 15-LOX that blocked ferroptosis in MCF-7, MDA-MB-231 breast cancer cells. It was shown that GSK-3β is a positive regulatory enzyme to promote cancer cell proliferation and survival and based on shreds of evidence, 15-LOX might be served by GSK-3β as a downstream mediator to regulate feroptosis in breast tumor cells [80]. On the other hand, it was shown that sodium butyrate as a short-chain fatty acid-induced 15-LOX gene expression and activity in breast cancer cells through elevation of 13-HODE [50]. The ability of sodium butyrate to trigger cell cycle arrest and apoptosis through the generation of ROS and reduced mitochondrial membrane potential (Δψm) in breast cancer cells [81] and its positive regulatory effect on 15-LOX may suggest that 15-LOX is contributed to the production of ROS in sodium butyrate-elicited apoptosis (Data is illustrated in Fig. 6). However, the significance of 15-LOX in the context of oxidative stress and redox biology in breast cancer pathogenesis is still emerging and needs to be clarified by more mechanistic studies.

## What are the clinical implications of 15-LOX inhibition or activation in breast cancer treatment?

The use of small molecular inhibitors that can inhibit key enzymes in the metabolic pathway is one of the main and important strategies in targeted cancer therapy [82]. Notably, interesting studies have been performed on small molecular compounds capable of inhibiting the 15-LOX, which can be considered as potent chemopreventive compounds in cancer prevention and treatment [83]. One of the well-documented ones is quercetin, which is a natural flavone with the ability to regulate cell cycle distribution, apoptosis, angiogenesis, and 15-LOX inhibitory activity [84]. It is demonstrated that quercetin sensitizes breast cancer cells to death and inhibits angiogenesis and EMT process, however, the efficiency of the quercetin-rich diet in breast cancer treatment needs to be clarified by further clinical trials [85]. Moreover, it was revealed that rhein and aloe-emodin, as polymethoxylated flavonoids which are extracted from orange peel inhibited soybean 15-LOX potently as well as breast cancer angiogenesis and growth [86]. On the other hand, Δ9 –tetrahydrocannabinol (Δ9-THC) [87], as an active component of *Cannabis Sativa* (*C. sativa*) and a member of phytocannabinoid family, can directly inhibit 15-LOX and suppress breast cancer cell proliferation [88]. The selective inhibitory effect of other derivatives of phytocannabinoids such as cannabidiol (CBD) on 15-LOX activity has been proven previously and in-vivo evidence revealed the potency of CBD to reduce breast cancer growth [88, 89]. The ortho-hydroxy analogs of pyrazoles have been shown to have an inhibitory effect on 15-LOX also the potential to inhibit breast cancer cell proliferation [90]. Taken together, the mentioned 15-LOX inhibitors showed anti-proliferative and cytotoxic effects on breast cancer cells through suppression of 15-LOX activity, however, the therapeutic use of these compounds in humans has not yet been addressed and requires further investigation at the clinical trial level. Evidence also has shown that 15-LOX-1 over-expression as well as exposure to the exogenous 13(S)-HODE enhanced drug accumulation, cell motility, subG1 arrest, apoptosis induction and, caspase 3/7 increased activity in doxorubicin-resistant MCF-7 cells, indicating the possibility of 15-LOX pathway involvement in the response of breast cancer cells to the doxorubicin treatment [46].

## Conclusion

This review attempts to highlight the expression pattern of 15-LOX enzymes and their metabolites in breast tumors and normal tissues as well as their significance in breast cancer cell growth, death, invasion, and metastasis. Studies show the dual role of the LOX pathway in the pathogenesis of breast cancer. Both decreased and increased expression of LOX enzymes were shown in breast tumor tissue compared to healthy breast tissues. Among the studies performed, it seems that 15-LOX-2 and 15-S-HETE showed a significant reduction in breast tumor tissue, which was adjusted by PPAR-γ activity. Based on the study of Hong et al., the expression pattern of 15-LOX isomers and metabolites in different types of breast cancer cells can be different which can explain the differential expression of these enzymes in different patients with breast cancer. Since in the patients studied more attention was paid to the degree of tumor severity, and disease severity and the molecular nature of breast tumor cells in these patients was not considered. On the other hand, the LOX enzymatic pathway is an important metabolic pathway for the normal life of healthy cells that can be affected by different conditions of growth, nutrition, and overall homeostasis of cells. Most of the results regarding the role of 15-LOX in inducing apoptosis in breast cancer cells indicated the mediating role of this enzyme or the synergistic effect of the metabolites of 15-LOX in inducing death pathways by other compounds. However, there was stronger evidence for the tumorigenic role of the 15-LOX pathway in breast tumor progression. It can be postulated that 15-LOX metabolites regulate breast tumor cell growth through elevation of cAMP level, phosphorylation of p38- MAPK, phosphorylation of ERK1/2, and increased tumor DNA content; however, the type of fat diet that the cells were exposed to, was decisive in the type of response they exhibited. It seems that 15-LOX-1 and its metabolites (15(S)-HETE 13(S)-HODE) were effective in breast cancer cell spheroids formation, metastasis to the lymph node, CREB activation, and transferring the mitogenic signals of TGF-α. Taken together, the relevance of 15-LOX enzymes and metabolites in breast tumor cell growth has been proven broadly; however, the exact role of this pathway on breast cancer pathogenesis remains to be determined, and future studies are warranted.

## Data Availability

All data generated or analyzed during this study are included in this published article.

## Abbreviations

ALA: Alpha-linolenic acid
LA: Linoleic acid
DHA: Docosahexaenoic acid
AA: Arachidonic acid (AA)
COX: Cyclooxygenase
PG: Prostaglandin
PGE: Prostaglandin E
TX: Thromboxane
TXA2: Thromboxane A2
CYP: Cytochrome P450
EETs: Epoxyeicosatrienoic acids
LT: Leukotrienes
LOX: lipoxygenase
HpETE: Hydroperoxyeicosatetraenoic acid
HETE: Hydroxyeicosatetraenoic acid
13-HODE: 13-Hydroxyoctadecadienoic acid
15-LOX: 15-lipoxygenase-15
15-LOX-1: 15-lipoxygenase type 1
15-LOX-2: 15- lipoxygenase type 2
A549 cell line: Adenocarcinoma human alveolar basal epithelial cells
Caco2 cell line: Human colorectal adenocarcinoma cells
IL4: Interleukin 4
IL13: Interleukin-13
STAT6: Signal transducer and activator of transcription 6
NO: Nitric oxide
15-S-HETE: 15(*S*)-hydroperoxyeicosatetraenoic acid
13(S)-HODE: 3(S)-hydroxy-9Z,11E-octadecadienoic acid
NSCLC: Non-Small Cell Lung Cancer
COX-2: Cyclooxygenase-2
CK19: Cytokeratin 19
ER: Estrogen receptor
PPAR-γ: Peroxisome proliferator- activated receptor gamma
PGE2: Prostaglandin E2
6-k-PGF1α: 6-keto-Prostaglandin F1 Alpha
PGF_2α_: prostaglandin F2α
HHT: Heptadecatrienoic acid
15-HETE: 15-Hydroxyeicosatetraenoic acid
DHKPG: 13,14-dihydro-l5-keto-prostaglandin
15-L-(S)-HPETE: 15-L-(S)-hydroperoxyeicosatetraenoic acid
COX-1: Cyclooxygenase-
5-LOX: 5-lipoxygenase
FLAP: 5-lipoxygenase activating protein
12-LOX: 12-lipoxygenase
ETYA: 5,8,11,14-eicosatetraynoic acid
NDGA: Nordihydroguaiaretic acid
ASA: Aspirin
9-HODE: 9-Hydroxyoctadecadienoic acid
13-HOTrE: 13S-hydroxy-9Z,11E,15Z-octadecatrienoic acid
9-HOTrE: 9-hydroxy-10E,12Z,15Z-octadecatrienoic acid
12-HHTrE: 12S-hydroxy-5Z,8E,10E-heptadecatrienoic acid
12-HETE: 12-Hydroxyeicosatetraenoic acid
5-HETE: 5-Hydroxyeicosatetraenoic acid
5-oxo-ETE: 5-Oxo-eicosatetraenoic acid
TSA: Trichostatin A
LA-enriched diet: High-fat diet enriched with linoleic acid
cAMP: Cyclic adenosine monophosphate
Erk1/2: Extracellular signal-regulated protein kinase 1/2
SR2: Steroid receptor negative
CLA: Conjugated linoleic acid
DMBA: 7,12-dimethylbenz [a]anthracene
PUFAs: Polyunsaturated fatty acids
MAPK: Mitogen-activated protein kinase
EPA: Eicosapentaenoic acid
MLT: Melatonin
AKT: Protein kinase B
pAKTs473: Phospho-Akt (Ser473)
NF-κB: Nuclear factor kappa B
CCID: Chemo repellent-induced defects
GLA: Gamma linoleic acid
MDSCs: Myeloid derived suppressor cells
CREB: cAMP Response Element-Binding Protein
BMEC: Bovine mammary endothelial cells
13-HPODE: 13-Hydroperoxyoctadeca-cis-9, trans-11-dienoic acid
TGF-α: Transforming growth factor alpha
EGF: Epidermal growth factor
ROS: Reactive oxygen species
GPX4: Glutathione peroxidase 4 (GPX4)
NFE2L2: nuclear factor erythroid-2 like 2
GSK-3β: Glycogen synthase kinase-3β
MDA: Malondialdehyde
Δψm: mitochondrial membrane potential
Δ9-THC: Δ9 –tetrahydrocannabinol
*C. sativa*: *Cannabis Sativa*
CBD: Cannabidiol
